# Report of Four Successful Operations, upon the Same Individual, for Stone in the Bladder

**Published:** 1868-04

**Authors:** J. C. Hughes


### REPORT OF FOUR SUCCESSFUL OPERATIONS UPON THE SAME INDIVIDUAL FOR STONE IN THE BLADDER.

Reported to the Iowa State Medical Society,

BY PROF. J. C. HUGHES, M. D.

GENTLEMEN: Permit me, as a member of this Society, to present for your consideration a brief report of a new and interesting fact connected with American Surgery. The subject to which I invite your attention is *Vesical Calculi*, in which Lithotomy has been practiced four times successfully by the Bilateral method.

Within the last eleven years I have been summoned at different times to visit Gen. Whitmore, an early settler of Iowa, and one of her most active and energetic citizens. This gentleman had for twenty years prior to my visit been a great sufferer from this disease, only finding relief from palliatives, and frequently by his own manipulations and the voiding of calculous deposit by the urethra.

In February, 1855, he requested me to visit him, and having given me an intelligent description of his condition, his intense sufferings, and his conviction that nothing could afford him relief save the knife, wished me to come prepared to operate. I accordingly visited him February 8th, and found the following symptoms present: Age, 62 years; general prostration; irregular thickening of the coats of the bladder; profuse muco-purulent discharge from the urethra, conjoined with the usual symptoms of *vesical calculi.* Conscious of his danger with or without an operation, and insisting upon the latter, I proceeded to operate by the bilateral method, removing *two calculi* of the triple phosphate variety.

His recovery from the operation, though under the care of an experienced practitioner, was not so rapid as I anticipated, requiring some thirty days, owing, doubtless, to the want of reparative power, due to his great age and enfeebled constitution. The case was now lost sight of for several months, the patient being once more enabled to resume the duties of his farm.

Again the symptoms of *calculi* so familiar to him, and which he so much dreaded, presented themselves, increasing in severity until he was again forced to seek surgical relief. In November, 1856, he visited me, requesting a second operation.

Upon examination into his condition and critically weighing the probabilities of recovery after *Lithotomy,* I decided upon the performance of Lithotripsy. Having prepared him for the operation by proper regimen and medication, in due time I introduced *Wiess' Lithotripster;* but the degree of suffering and prostration created by the use of the instrument obliged me to desist after two ineffectual efforts to crush the calculus.

On the 29th of November, several days after the attempt at Lithotripsy, I performed the *bilateral* operation of *Lithotomy* before the Class of the Medical Department of the Iowa State

University, removing a *calculus* of large size, and of the same variety as the former.

The patient rapidly recovered from the operation, the wound being entirely closed on the twelfth day, and the patient able to leave his room. I now placed the patient under a strict dietetic course, coupled with such remedies as were deemed proper to correct the existing diathesis. Soon forgetting his troubles, he abandoned all treatment, and returned to his accustomed avocation.

In January, 1858, I received a note from Dr. Ware, stating that the General was again suffering with former symptoms, and was convinced that he had discovered another *calculus;* was anxious to visit me, but was unable to do so. Just at this time I was preparing to visit our State capital, and *en route* would pass within a few miles of my patient. I replied to Dr. Ware to that effect, and if agreeable to the General, would stop over and visit him. Not knowing but that a third operation might become necessary, I took from my operating case the double *Lithotomie Cachœ*, of Dupaytren, a sound, staff, one pair of short forceps, and a pocket case. Armed in this way I proceeded on my journey, and at the county seat of Jefferson was met by a messenger requesting me to visit the General.

Next morning, in company with Drs. Ware, Hewey, Steele, Shafer, Walker and others, I called upon the patient, found him emaciated and much reduced in strength, and in a condition more unfavorable for a successful operation than at any former period, yet very desirous of again undergoing the ordeal. After free consultation we decided to accede to his wish, and at once proceeded to perform the *bilateral operation*, and without difficulty succeeded in removing *three calculi.* I then discovered a fourth, which proved to be encysted, having for its site the fundus of the bladder. With the short forceps I made several fruitless attempts at its removal, and only by prolonged and laborious efforts was I enabled to secure it.

Having cleansed thoroughly the parts, and the patient once more comfortable, I left him in charge of Dr. Ware, almost without hope of his recovery. Much to our surprise the case progressed favorably, but restoration to health more tedious than after former operations.

A year had scarcely passed until he was again conscious of the presence of *vesical calculi.* To extreme suffering and old age his shattered frame almost succumbed, and for near a twelve month he was confined to his room, suffering at frequent intervals the most intolerable agony from this terrible malady.

On the 23d April, 1860, I received a note from his medical attendant to the effect that the General wished me to come immediately and operate upon him. Imagine my surprise at this extraordinary summons. I at once obeyed, and on the 26th of April performed the fourth operation bilateral, removing *twelve calculi.*

He soon rallied, and I left him with more hope of recovery than in former operations. Twenty-four hours afterwards his physician, Dr. Ware, wrote me as follows:

Dr. Hughes—*Dear Sir:* I visited Gen. Whitmore this day at 9 A. M. Found him comfortable; no febrile action; pulse 78 and regular; skin moist; urine looking better than usual, a little tinged with blood, but little mucous; slept but little; no pain; took a little toast; said he had not felt so comfortable in many months; felt decidedly better this time than after any previous operation. The cut looks healthy; no angry appearance; no soreness, and in fact I have not seen the General so cheerful in a long time.

Yours, truly, J. C. Ware.

The Doctor again writes, May 1st, as follows:

"I visited the General Monday morning at 9 o'clock, four days subsequent to the operation. Found him comfortable, with the exception of some uneasiness in his bowels; no fever; pulse 75; skin in good condition; tongue moist, and of healthy color; had rested pretty well; spirits cheerful; cut looks healthy; suppurating a little; discontinued cold applications; had taken some fresh fish; ordered cathartic, to be followed, if necessary, by an injection; as diet, beef tea, rice, &c. The General doos not deem it necessary for me to return, unless sent for. Yours, &c., J. C. Ware."

The only letter received from the Doctor since was dated May 20th, in response to an inquiry, in which he says :

"I have visited the General but three times, twice having given you the particulars as far as noted. At my last visit, some twelve or fourteen days after I last wrote you, I found the cut had healed, having given him no trouble, being entirely closed in about twelve days after the operation. He has been up and walking around, his general health good."

This case presents many interesting features. Its locality is in the wooded district, whose geological formation consists of drift deposit coal and different qualities of limestone. The water used is found in the drift, which consists of beds of clay, gravel and sand, strongly impregnated with lime, and is furnished from underground streams that percolate through its sandy portions. Whether the disposition to the formation of calculi should be attributed to the geological character of the locality I do not pretend to determine. Such was the uncontrolable wilfulness of the patient, that neither regimen, nor medication recommended for the change of diathesis, could be enforced or persevered in for a sufficient length of time to test their utility. I am inclined to ascribe Gen. Whitmore's rapid recovery from so frequent operations, not only to his constitutional vigor and indomitable will, but in no small degree to the *mode of operation*, which I claim to be safer to the patient, performed with greater facility, and followed by more expeditious recovery, than from the *lateral method.* It is now six years since I last operated on the General. He is yet alive, and, although at such an advanced age, is in the enjoyment of comparatively good health.

I was first induced to adopt the *bilateral* mode of operation in 1853, upon a patient whose *rectum* had been cut into during a previous operation by the *lateral method.* Such were the adhesions which then existed between the rectum and bladder that I considered this mode the safest under the circumstances, as thereby I would more certainly avoid any injury to the rectum, which would almost inevitably ensue were I to cut into or near the former incision. This was the first *bilateral* operation performed in the State, and I confess that the ease and beauty of this mode, and the rapidity of the recovery in the case, did much towards removing from my mind the prejudices I formerly entertained against it. The cut from within outwards is a smoother one, the danger of infiltration less, and more likely to unite by first intention, the risk to the rectum not so great, a larger opening with less risk of wounding the pudic arteries, and last, though not least, an easier mode of operating.

Having performed the *bilateral* operation in twenty-one cases, with the loss of but a single case, I think I am justified in advocating its claims in preference to the *lateral* mode, as against the opinion of more experienced Surgeons.

Believing that I have submitted to the Iowa State Medical Society an account of the only instance in which *four* successful operations for removal of urinary calculi have been performed upon the same patient, either in America or Europe, I am anxious that whatever credit may redound therefrom to American Surgery, shall be secured by its publication in the transactions of this Society.

